# Engaging women and men in the gender-synchronised, community-based Mbereko+Men intervention to improve maternal mental health and perinatal care-seeking in Manicaland, Zimbabwe: A cluster-randomised controlled pragmatic trial

**DOI:** 10.7189/jogh.12.04042

**Published:** 2022-05-21

**Authors:** Liz Comrie-Thomson, Karen Webb, Diana Patel, Precious Wata, Zivanai Kapamurandu, Angela Mushavi, Mary-Ann Nicholas, Paul A Agius, Jessica Davis, Stanley Luchters

**Affiliations:** 1 Burnet Institute, Melbourne, Australia; 2 Department of Public Health and Primary Care, Ghent University, Ghent, Belgium; 3 Department of Epidemiology and Preventive Medicine, School of Public Health and Preventive Medicine, Monash University, Melbourne, Australia; 4 Faculty of Medicine, Dentistry and Health Sciences, University of Melbourne, Carlton, Australia; 5 Organization for Public Health Interventions and Development, Harare, Zimbabwe; 6 Envision Zimbabwe Women’s Trust, Harare, Zimbabwe; 7 Regional PsychoSocial Support Initiative (REPSSI) Zimbabwe, Harare, Zimbabwe; 8 National PMTCT Program, AIDS & TB Unit, Ministry of Health and Child Care, Harare, Zimbabwe; 9 Independent consultant; 10 Centre for Sexual Health and HIV/AIDS Research (CeSHHAR), Harare, Zimbabwe; 11 Liverpool School of Tropical Medicine, Liverpool, UK

## Abstract

**Background:**

Maternal mental morbidity and low perinatal health service utilisation in resource-constrained settings contribute substantially to the global burden of poor maternal, newborn, and child health. The community-based Mbereko+Men program in rural Zimbabwe engaged women and men in complementary activities to improve men’s support for women and babies, coparents’ equitable, informed health decision-making, and ultimately, maternal mental health and care-seeking for maternal and newborn health services. The study aimed to test the effectiveness of the Mbereko+Men program on maternal mental health at 0-6 months after childbirth.

**Methods:**

We conducted a cluster-randomised controlled pragmatic trial using a two-arm parallel design with four clusters per arm. Data was data collected through cross-sectional surveys before and after the implementation of the intervention or standard care. Rural health facility catchments in Mutasa District, Zimbabwe, were randomised using a true random number sequence. Survey participants were women who had given birth within 0-6 months and their male coparents. The primary outcome was women’s mean Edinburgh Postnatal Depression Scale (EPDS) score. Secondary outcomes captured care-seeking, men’s supportive behaviours, and gender dynamics in coparent relationships. Masking was not used. All clusters were included in the analysis. The trial was registered with the Australian New Zealand Clinical Trials Registry (ACTRN12620001014943) in October 2020.

**Results:**

Between April 13 and May 20, 2016, 457 women and 242 men participated in the pre-intervention survey; between October 19 and November 30, 2017, 433 women and 273 men participated in the post-intervention survey. Women’s mean EPDS scores declined in both arms. The decline was 34% greater in the intervention arm (adjusted risk ratio = 0.66; 95% confidence interval = 0.48, 0.90, *P* = 0.008). Improvements in care-seeking, men’s support, and coparents’ relationships were detected.

**Conclusions:**

A low-intensity gender-synchronised intervention engaged women and men to improve maternal mental health and care-seeking in a setting characterised by gender inequality and demand-side barriers to care.

In the year following childbirth, one in five women in low- and middle-income countries is affected by common perinatal mental disorders, typically depression and anxiety, [1]. Maternal mental morbidity makes it more difficult for women to care for themselves and their children, or engage with health services, and contributes to adverse obstetric outcomes and constrained child growth and development [2]. Risk factors for maternal mental morbidity include high-conflict, low-empathy relationships between coparents and receiving low levels of practical support during pregnancy and the first year after childbirth [1,2].

There are effective approaches to improve maternal mental health, care-seeking for maternal, newborn and child health (MNCH) services, and male coparents’ practical support for women and babies. Women’s participatory learning and action (PLA) groups and interventions to influence men’s engagement in MNCH have separately been found to improve maternal mental health, care-seeking, and maternal and neonatal mortality [3-6], while interventions targeting coparents together can improve mutual support and maternal mental health [7]. Gender-synchronised interventions target women and men with separate but complementary messages and can be an effective approach for addressing interrelated, gendered causes of poor MNCH outcomes [8,9].

This study aimed to test the effectiveness of a gender-synchronised, low-intensity, community-based intervention – Mbereko+Men – to improve maternal mental health and MNCH care-seeking in a resource-constrained setting. The intervention was designed to achieve change through two interrelated mechanisms: coparents’ informed and equitable MNCH decision-making, and men’s practical support for women and babies (**Figure 1**). We hypothesised that, through these mechanisms, the intervention would both improve maternal mental health and increase optimal care-seeking for essential MNCH services (**Figure 1**, **Table 1**).

**Figure 1:**
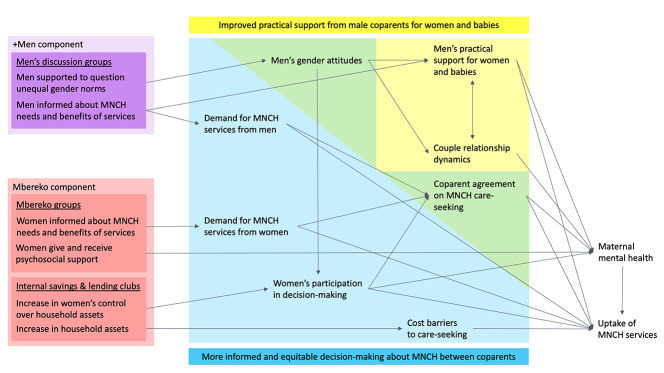
Mbereko+Men intervention theory of change. MNCH – maternal, newborn, and child health.

**Table 1:** Study outcomes and measures

Outcome of interest	Measure	Respondents	Items	Range and interpretation	Hypothesised direction of change	Cronbach’s alpha
Symptoms of depression and anxiety	EPDS	Women	10 items scored 0-3	0-30, higher score indicates more and/or more frequent symptoms	Lower scores	0.87 [10]
Uptake of essential MNCH services	MNCH care-seeking	Women	6 items scored 0/1, reported separately	0/1 per item, score of 1 indicates optimal uptake of the service	Higher scores	n/a
Women’s participation in decision-making	Women’s Household Decision-making Power Scale	Women	3 items scored 0/1, reported (a) separately and (b) summed	(a) 0/1 per item, score of 1 indicates woman makes the decision alone or together with another person (b) 0/3, score of 3 indicates the woman is involved in making all three decisions (vs none of the decisions)	Higher scores	0.92 [11]
Men’s gender attitudes	Gender Equitable Men Scale	Men	22 items scored 1-3	22-66, higher score indicates more inequitable attitudes	Lower scores	0.81 [12]
Couple relationship dynamics	Intimate Bond Measure	Women	24 items scored 0-3, comprising two sub-scales (Care and Control) each with 12 items	0-72 (sub-scales 0-36), higher score indicates woman’s partner is less caring and/or more controlling	Lower scores	Care sub-scale = 0.79-0.83, Control sub-scale = 0.68-0.79 [13]
Men’s practical support for their female coparent and baby	Male coparent practical support	Men	15 items scored 0/1, reported separately	0/1 per item, score of 1 indicates man provides at least some support	Higher scores	n/a

## METHODS

### Study design

The study was a cluster-randomised controlled pragmatic trial, using a two-arm parallel design with four clusters per arm. The study arms comprised no intervention (standard care) and full intervention clusters. The unit of randomisation was the health facility catchment area. Intervention effects were estimated by comparing outcome measures between participants sampled independently before and after implementation of intervention or standard care, using a study arm by time (pre- and post-intervention) interaction term.

### Study sites and participants

Women, newborns, and children at the study site in Mutasa District, Zimbabwe, face an unacceptably high burden of poor health, with an estimated infant mortality rate of 87 deaths per 1000 live births (2011 data) [14]. At provincial level, 30.6% of children are stunted and uptake of MNCH services is low, with births outside health facilities (25.5% of all births) and missed childhood vaccinations (12.9% of children aged 12-23 months receiving no vaccinations) the highest in the country [15]. Barriers to care-seeking include unequal gender norms – which limit women’s participation in decision-making and access to household resources, and discourage men from supporting MNCH – as well as limited social support for women, financial resource constraints, and low awareness of the benefits of MNCH services [16,17].

Catchment areas of Rural Health Centres or Rural Hospitals in Mutasa District were eligible for inclusion in the study if there was no concurrent non-government MNCH program supplementing standard care in the catchment. Antenatal care attendance records for April-September 2014 were used to identify and exclude sites with the lowest estimated birth rates. Sites were excluded if they adjoined an already included site.

Community-based surveys were aimed to achieve complete enumeration of eligible women and men pre- and post-intervention. First, eligible women participants were identified in consultation with village health workers through community meetings targeting all parents of young children. This was followed by snowball sampling to identify eligible women who had not attended the meetings. Women were eligible to participate in the study if they were aged 16 years or older, had resided at the study site for at least 12 months, had given birth within the previous six months, and provided written informed consent. Once a woman was enrolled in the study, field researchers sought her verbal informed consent to invite the male coparent of her child aged 0-6 months to participate in the study. Field researchers then approached the man identified by the enrolled woman as her coparent, to assess his eligibility. Men aged 16 years and over, who had resided at the study site for at least 12 months, whose enrolled female coparent provided verbal consent for their participation, and who themselves provided written informed consent were eligible. Coparents were defined as the biological mother and either her current male partner or the man she identified as the father of her child. Relationship status, marital status, and biological fatherhood were not considered inclusion or exclusion criteria.

### Intervention

The Mbereko+Men intervention was delivered through two gender-synchronised components implemented separately with women and men. Components were delivered at the cluster level through community-based training and discussion groups and were intended to achieve a community-level effect. All women who were pregnant or had a child aged up to two years and all men residing in intervention sites were invited to participate in intervention activities. Intervention components were delivered over 12-15 months, depending on the cluster, with the intervention progressively scaled up to all four intervention clusters over three months.

The first intervention component (Mbereko) was delivered by trained local female village health workers, supervised by a trained female project staff member with over three years of experience in facilitating women’s PLA groups. Women participated in PLA cycles conducted through monthly one-hour group discussions, facilitated by village health workers in a central community location. Discussions explored MNCH services and home care practices recommended during pregnancy and between zero and two years of age, including services for the prevention of mother-to-child transmission of HIV (PMTCT). Facilitators used flip charts and group discussions to share health information and discuss recommended actions. Facilitators supported women through PLA cycles grounded in problem-solving therapy [18] to first identify barriers to following recommended actions, then plan to overcome these barriers with available resources and subsequently reflect on their experiences. Each woman was provided with an Action Birth Card, a handheld information and goal-setting tool, to support her planning and reflection [19]. Discussion groups were integrated with internal savings and lending clubs, delivered through five two-hour training sessions and subsequent meetings at club members’ discretion, where small clubs formed from discussion group members pooled their savings and jointly decided on how to invest income-generating activities and manage interest and repayments.

Mbereko groups have been implemented in rural Zimbabwe by the Organization for Public Health Interventions and Development (OPHID) since 2010 [16]. Building upon local evidence that rural women in Zimbabwe value male partner support [17,20], this study introduced a second intervention component (+Men). The +Men component was delivered by a trained male OPHID staff member who was also a nurse and midwife with substantial community development experience, targeting men residing in the same communities where Mbereko groups were established. Men participated in monthly one-hour group discussions, facilitated by the male project staff member in men’s workplaces or a central community location. Discussions explored similar health topics to those addressed in women’s groups and the same flip chart was used to present information; topics in women’s and men’s groups were sequenced so that women’s insights on gender-related challenges relevant to each topic could inform how the topic was subsequently discussed with men. Groups also completed facilitated reflections on norms underpinning the gendered division of labour in domestic and care work, safe sex during pregnancy, and men’s contributions to MNCH care-seeking. Each men’s group incrementally developed a participatory Men’s Charter for Family Health [21], using facilitated discussion at the conclusion of each monthly meeting to identify and commit to principles and behaviours to improve their coparents’ and children’s health. After 12-15 months, completed charters were presented to men for public display in their community.

To support the Mbereko and +Men components, project staff ran two-hour Health Centre Committee meetings monthly for 12-15 months, bringing together community leaders and health facility staff to review clinic performance and community health outcomes and strengthen community-facility linkages.

### Randomisation

The Mbereko+Men intervention was randomised (1:1) at the cluster level by an external researcher who had no other involvement in the trial, using a true random number sequence (online random number generator, www.random.org). Masking was not used.

### Study procedures

Two independent samples of participants in intervention and control sites were taken before and again after 12-15 months of intervention implementation. Enrolled participants had a structured questionnaire administered by trained field researchers of the same gender who had current Good Clinical Practice (ICH-GCP) certificates. Separate questionnaires were prepared for men and women participants. Questionnaires were developed in English, except for the primary outcome measure which had been previously validated in Shona [10], the main language spoken by the study population. Questionnaires were translated to Shona and back-translated to English by two native Shona speakers fluent in English. Field researchers were native Shona speakers and administered the questionnaires in Shona. No field researchers who administered the questionnaires post-intervention were involved in delivering the intervention. Questionnaires were administered with visual and auditory privacy, either at a communal location, in participants’ homes, or over the phone for male participants who were absent from study sites during field data collection. Phone interviews were used in the post-intervention survey only, in response to challenges in reaching men during the pre-intervention survey. Additionally, completed Men’s Charters for Family Health developed in intervention sites were recorded. After the post-intervention survey, the intervention was implemented in control sites.

The study was retrospectively registered in the Australian New Zealand Clinical Trials Registry (ACTRN12620001014943) on 7 October 2020. A data monitoring committee did not oversee the study. All study participants provided written informed consent prior to their enrolment in the study, with those aged 16-17 years independently consenting as emancipated minors under national regulations.

### Outcome measures

All primary and secondary outcome measures (**Table 1**) were defined *a priori* in the study protocol, approved by the Medical Research Council of Zimbabwe on 28 March 2016 and the Alfred Research Ethics Committee on 13 January 2016. The primary outcome measure was women’s mean score on the locally validated [10] Shona-language version of the Edinburgh Postnatal Depression Scale (EPDS), a 10-item scale yielding scores ranging 0-30, with higher scores indicating more, or more intense, symptoms of depression and anxiety [22]. One secondary outcome measure was the proportion of women meeting the EPDS cut-off of 11/12, locally validated as indicating clinically significant symptoms of depression and anxiety [10].

Additional secondary outcome measures were selected to align with the intervention’s hypothesised theory of change (**Figure 1**). *Health care-seeking* for essential MNCH services was measured using the proportion of women who for their most recent pregnancy commenced antenatal care in the first trimester, attended four or more antenatal care visits, gave birth in a facility, and reported timely postnatal care for themselves (0-6 weeks from birth) and their newborn (0-72 hours from birth), as well as the proportion of women reporting both they and their current male partner had completed an HIV test during their most recent pregnancy. *Women’s participation in decision-making* was measured as the proportion of women involved in each, all, and none of three categories of household decisions (decisions about major purchases, visits to family, and care-seeking for the woman’s health) [23]. *Men’s gender attitudes* were assessed using men’s mean score on the Gender Equitable Men scale, adapted by adding an item dropped during validation but recommended in the validation study for use when relevant (“Men can take care of children just as well as women can”) [12]. *Couple relationship dynamics* were assessed using women’s mean scores on the validated Intimate Bond Measure, designed to capture respondents’ experiences of their current partner’s behaviour through two individually validated sub-scales measuring partners’ emotionally supportive (Care sub-scale) and controlling (Control sub-scale) behaviours [13]. Finally, *male coparent practical support for women and babies* was assessed as the proportion of men providing instrumental and financial support across 15 items, developed for this study based on existing measures [5,13] and domains identified through previous qualitative work in rural Zimbabwe [17]. Each support item was modelled as a binary variable for analysis, either yes vs no or sometimes/usually vs never.

Plausible adverse events related to increased tension in coparent relationships, resulting from changed expectations and behaviours [5]. These were assessed through women’s physical intimate partner violence victimisation using the World Health Organization validated measure [24], men’s and women’s experiences of controlling behaviour perpetrated by their partner using mean score on the validated Intimate Bond Measure Control sub-scale [13], and acceptability to women of support provided by their male coparent using the validated Social Support Effectiveness measure [25].

### Statistical analysis

For the primary outcome, linear modelling was used to estimate that a sample size of 880 women (110 women per health facility catchment area, divided between pre- and post-intervention time points) was needed to detect a difference in means [*b*] of approximately 2.75 points in EPDS score (small/moderate effect size, Cohens d = 0.38) between intervention and control groups, assuming a standard deviation in EPDS of 7.9 as previously found in Zimbabwe [10], 5% significance, 80% power, and intracluster correlation of 0.02. However, given the non-normal distribution of EPDS scores in the data, linear modelling was excluded, and generalised linear modelling (log link function and negative binomial distribution) was instead used to estimate the intervention effect on mean EPDS, with a study arm by time (pre- and post-intervention) interaction term estimated in addition to main effects for time and study arm. To account for inflation in standard error due to cluster randomisation, clustered sandwich estimator was used to estimate cluster robust standard errors. The same model structure was applied to estimate intervention effects and standard errors for secondary and exploratory analyses, with a logit link function and binomial distribution assumed for categorical variables, and ordinary least squares regression modelling undertaken for continuous variables. Exploratory analyses, which were not prespecified, estimated intervention effects and standard errors for women’s mean scores on the Intimate Bond Measure Care and Control sub-scales, in addition to the prespecified analysis of women’s mean Intimate Bond Measure total score. Models were adjusted for age, education, and number of pregnancies (for women) or number of children (for men). A random-effects ANOVA model was used to calculate intracluster correlation coefficients for the primary outcome.

Analysis was conducted in Stata 15.1. For each analysis, participants with any missing data for variables of interest were excluded.

### Ethics

Ethical approval was granted by the Medical Research Council of Zimbabwe (MRCZ/A/2006) and Alfred Research Ethics Committee (394/15). The approved study protocol is available online (https://www.burnet.edu.au/system/asset/file/4759/Mbereko%2BMen-study-protocol.pdf).

## RESULTS

**Figure 2:**
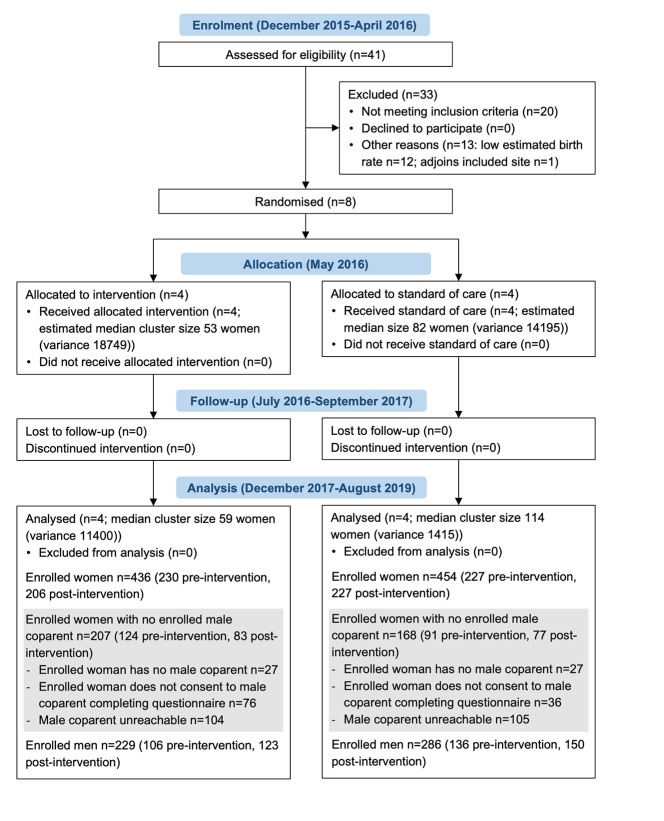
Participant flow diagram. Number of women and men who declined to complete the questionnaire not recorded.

After applying eligibility criteria, eight health facility catchment areas (clusters) were identified: Mt Jenya Clinic, Old Mutare Mission Hospital, Sadziwa Clinic, Sherukuru Rural Health Centre, St Augustine’s Clinic, St Barbara’s Mission Hospital, Triashill Mission Hospital, and Zongoro Clinic. Four clusters were randomly assigned to the intervention arm, and four to the control arm. There were no losses or exclusions after randomisation at cluster or individual level in either intervention or control arms (**Figure 2**). In intervention sites, activities were progressively rolled out from July to September 2016, and delivered until September 2017. Over this period of 12-15 months, a total of 35 Mbereko women’s groups (comprising 634 women) and 30 men’s discussion groups (with 781 men attending) held monthly sessions. Men’s Charters for Family Health were generated by all 30 men’s discussion groups between October 4, 2016, and September 28, 2017.

Participants were enrolled at all clusters for data collection pre-intervention, between April 13 and May 20, 2016, and again post-intervention between October 19 and November 30, 2017. No intervention activities were conducted in control sites until data collection concluded. The questionnaire was completed by 436 women and 229 men in the intervention arm, and 454 women and 286 men in the control arm, divided between pre- and post-intervention time points (**Figure 2**). No participant refusals were recorded. Based on 2014 antenatal care enrolments at included health facilities and 2019 data on coverage of antenatal care in Mutasa District [15], an estimated 78% of women who had given birth 0-6 months before the survey (74% intervention, 82% control) were sampled. A minority of enrolled women reported they did not have a male coparent or declined consent for their male coparent to be enrolled in the study (**Figure 2**). Field researchers were able to reach 71% of men whose female coparents consented for them to complete the questionnaire (69% intervention, 73% control).

Participants’ baseline socio-demographic characteristics were similar between study arms (**Table 2**). Men’s mean age, men’s number of children, and the proportion of women not in a relationship were higher in the intervention arm. No woman in the control arm and two (0.9%) women in the intervention arm had had their most recent pregnancy result in a stillbirth.

**Table 2:** Baseline characteristics by study arm*

	Control	Intervention
Clusters, number participating	4	4
Average number of women (total pre- and post-intervention)	114 (62)	59 (111)
**Women, number participating pre-intervention**	227	230
Age (years)	25.1 (6.6)	25.8 (6.7)
Completed primary school	182 (80.2%)	171 (74.3%)
Engaged in paid work	96 (42.3%)	92 (40.0%)
**Reproductive history**		
Number of children	2.4 (1.4)	2.4 (1.4)
Number of pregnancies	2.5 (1.5)	2.7 (1.7)
Most recent pregnancy resulted in stillbirth	0/226 (0.0)	2 (0.9)
**Relationship status**		
Married – monogamous	204 (89.9%)	198 (86.0%)
Married – polygamous	13 (5.7%)	16 (7.0%)
In a relationship, not married	2 (0.9%)	1 (0.4%)
Not in a relationship	8 (3.5%)	15 (6.5%)
Married before 18	82/212 (38.7%)	74/214 (34.6%)
Male partner usually lives in woman’s household	185 (81.5%)	186 (80.9%)
**Men, number participating pre-intervention**	136	106
Age (years)	31.8 (7.7)†	35.2 (8.6)
Completed primary school	103 (79.3%)	84/105 (80.2%)
Engaged in paid work	85/135 (62.6%)	73 (68.5%)
Number of children	2.5 (1.5)	3.0 (2.1)

*Data are n (%), n/N (%), median (interquartile range), or mean (standard deviation).

†Age data available for 135/136 participants.

From pre- to post-intervention, women’s mean EPDS score declined by 63% in the intervention arm (adjusted rate ratio (aRR) = 0.37; 95% confidence interval (CI) = 0.26, 0.51, *P* < 0.0001; intracluster correlation coefficient (ICC) = 0.01) and 45% in the control arm (aRR = 0.55; 95% CI = 0.48, 0.63, *P* < 0.0001; ICC = 0.00) adjusted for individual women’s age, gravidity and educational attainment, and clustering effects. The decline in mean EPDS was 34% greater in the intervention arm compared to the control arm (aRR = 0.66; 95% CI = 0.48, 0.90, *P* = 0.008; ICC = 0.00) (**Table 3**).

**Table 3:** Women’s mental health, care-seeking, and participation in decision-making*

	Control		Intervention		Effect	*P*-value†
	**Pre-intervention**	**Post-intervention**	**Pre-intervention**	**Post-intervention**		
**Primary outcome**					aRR (95% CI)	
EPDS	7.2 (0.2)	4.0 (0.3)	8.0 (0.2)	3.0 (0.4)	0.7 (0.5- 0.9)	0.008
**Secondary outcomes**					aOR (95% CI)	
Mental health						
EPDS≥12	51/225 (22.7%)	18/219 (8.3%)	61/225 (27.1%)	19/199 (9.8%)	1.0 (0.6- 1.4)	0.810
**Care-seeking for MNCH services**						
Timely ANC (first trimester)	92/222 (41.4%)	84/204 (41.8%)	89/225 (39.6%)	106/200 (52.8%)	1.7 (1.1, 2.6)	0.015
4 or more ANC visits	157/223 (70.4%)	158/221 (71.1%)	170/228 (74.6%)	151/201 (74.5%)	1.0 (0.4, 2.4)	0.938
Couples HIV test in pregnancy	177/223 (79.4%)	137/213 (64.0%)	167/222 (75.2%)	146/196 (73.8%)	2.1 (1.3, 3.4)	0.003
Facility birth	204/224 (91.1%)	196/212 (92.3%)	200/224 (89.3%)	187/198 (94.3%)	1.9 (0.6, 5.9)	0.257
Timely postnatal care (mother)	205/220 (93.2%)	187/201 (93.0%)	202/218 (92.7%)	194/195 (99.5%)	15.7 (5.4, 45.3)	<0.0001
Timely postnatal care (baby)	32/221 (14.5%)	22/199 (11.2%)	34/219 (15.5%)	55/195 (27.9%)	2.8 (1.7, 4.8)	<0.0001
**Participation in decision-making**						
Major household purchases	134/224 (59.8%)	154/226 (67.7%)	148/226 (65.5%)	165/205 (81.0%)	1.6 (0.9, 2.9)	0.144
Visits to family	166/225 (73.8%)	169/225 (74.8%)	159/225 (70.7%)	177/202 (87.4%)	2.7 (1.8, 4.2)	<0.0001
Health visits for woman	164/225 (72.9%)	181/224 (80.5%)	157 (68.3%)	172/205 (84.0%)	1.6 (0.6, 4.2)	0.356
All three decisions	93/224 (41.5%)	118/225 (51.8%)	93/224 (41.5%)	138/203 (68.7%)	2.0 (1.2, 3.5)	0.012
None of the decisions	20 (8.8%)	20/224 (9.0%)	20 (8.7%)	11/205 (5.5%)	0.6 (0.3, 1.3)	0.176

aRR – adjusted rate ratio. aOR – adjusted odds ratio. ANC – antenatal care. EPDS – Edinburgh Postnatal Depression Scale.

*Data are n (%), n/N (%), or mean (standard error), with variance estimation accounting for health facility catchment clustering. Probability values from Wald χ^2^ tests based on cluster robust standard errors. aRR and aOR reflect time by intervention group interactions, adjusted for individual women’s age, gravidity, and educational attainment.

The change in the proportion of women with clinically significant symptoms of depression and anxiety (EPDS≥12) was not significantly different between the intervention and control arms (**Table 3**).

Care-seeking increased in the intervention arm relative to the control arm across four measures: 1) proportion of women attending antenatal care in the first trimester, 2) proportion of women receiving postnatal care within six weeks after childbirth, 3) proportion of newborns receiving postnatal care within 72 hours after birth, and 4) proportion of couples receiving an HIV test during pregnancy (**Table 3**). There was no effect detected on the proportion of women who had four or more antenatal care visits or facility birth.

The proportion of women participating in household decision-making about visits to family increased in the intervention arm, as did the proportion of women participating in decision-making about all three topics measured (**Table 3**). There was no effect detected for women’s participation in decision-making about their health visits or major household purchases, or the proportion of women not involved in making decisions about any of the three topics.

Men’s gender attitudes were less inequitable, and couple relationship dynamics were less harmful, in the intervention arm (**Table 4**). Exploratory analyses of Intimate Bond Measure sub-scales found no effect on women’s Care sub-scale mean score, while women’s Control sub-scale mean score was lower in the intervention arm, indicating less controlling behaviour by male partners.

**Table 4:** Couple relationship dynamics and men’s gender attitudes*

	Control		Intervention		*b* (95% CI)	*P*-value†
	**Pre-intervention**	**Post-intervention**	**Pre-intervention**	**Post-intervention**		
**IBM total score**	27.3 (1.4)	23.0 (1.5)	29.0 (0.9)	21.2 (0.6)	-3.4 (-6.7, -0.0)	0.048
**IBM Care sub-scale score**	6.5 (0.6)	4.8 (0.3)	6.8 (0.3)	5.2 (0.2)	0.1 (-1.5, 1.7)	0.858
**IBM Control sub-scale score**	20.9 (0.8)	18.3 (1.3)	22.2 (0.7)	15.9 (0.8)	-3.6 (-6.8, -0.5)	0.030
**GEM score**	36.5 (0.3)	37.2 (0.3)	35.5 (0.6)	34.0 (0.7)	-2.4 (-3.8, -1.0)	0.005

IBM – Intimate Bond Measure. GEM – Gender-Equitable Men.

*Data are mean (standard error), with variance estimation accounting for health facility catchment clustering. Probability values from t-distributions based on cluster robust standard errors. *b* reflects a time by intervention group interaction for the difference in means, adjusted for individual participants’ age, gravidity (for women) or number of children (for men), and educational attainment.

The proportion of men reporting they provided practical support to their female coparents and babies during pregnancy, childbirth and after birth increased in the intervention arm across eight out of 15 measures (**Table 5**). More men in the intervention arm participated in MNCH services by accompanying their coparent to antenatal care or the place of childbirth, being present during childbirth, or taking their sick baby to a health facility. Men in the intervention arm increased their support for breastfeeding, as well as maternal nutrition during pregnancy or postpartum. More men in the intervention arm engaged in baby care, such as bathing or playing with the baby. No effect was detected on the proportion of men participating in antenatal care consultations, supporting childbirth by providing money or goods, contributing to household chores during pregnancy or after childbirth, encouraging their pregnant coparent to rest, or settling their baby at night.

**Table 5:** Men’s practical support for women and babies*

	Control		Intervention		aOR (95% CI)	*P*-value
	**Pre-intervention**	**Post-intervention**	**Pre-intervention**	**Post-intervention**		
**Support during pregnancy**†						
Obtain or prepare special foods for woman	129 (96.5%)	139 (93.8%)	101 (94.5%)	118 (96.6%)	2.9 (1.1, 7.5)	0.031
Encourage woman to rest	127 (93.9%)	141 (95.1%)	103 (96.7%)	119/122 (97.4%)	0.9 (0.2, 3.6)	0.918
Contribute to household chores	127/134 (95.6%)	136 (91.7%)	95 (87.9%)	116 (94.0%)	4.2 (1.0, 18.1)	0.056
Accompany woman to ANC	95/134 (72.6%)	86/146 (59.6%)	69 (63.7%)	97 (78.6%)	4.2 (1.7, 10.5)	0.002
Participate in ANC consultation	78/135 (59.6%)	60/147 (41.8%)	54/104 (52.8%)	65 (52.1%)	2.1 (0.8, 5.0)	0.110
**Support around childbirth**‡						
Accompany woman to place of childbirth	84/135 (67.5%)	83/141 (58.4%)	71 (67.0%)	95/122 (78.4%)	2.7 (1.5, 4.7)	0.001
Present during childbirth	17 (12.2%)	14/149 (9.8%)	16/104 (16.7%)	36 (29.9%)	2.7 (1.1, 7.1)	0.039
Financial support for childbirth	128/134 (97.3%)	139/148 (93.7%)	101 (96.7%)	120 (97.4%)	3.3 (0.3, 32.0)	0.306
Provide baby clothes or other items for childbirth	133/135 (99.1%)	148 (98.6%)	105 (98.9%)	121 (98.3%)	1.0 (0.0, 21.9)	0.983
**Support for woman after birth**†						
Contribute to household chores	122/135 (91.3%)	127/146 (87.9%)	86/103 (83.0%)	108/121 (88.7%)	2.4 (0.8, 6.9)	0.109
Encourage woman to breastfeed	129/135 (94.8%)	142/148 (96.5%)	95/103 (90.9%)	120/121 (99.1%)	8.0 (2.0, 32.4)	0.004
Obtain or prepare special foods for woman	124/135 (93.0%)	123/147 (85.1%)	94/103 (90.9%)	118/121 (97.4%)	9.2 (3.8, 22.1)	<0.0001
**Support for baby care**						
Bathe, dress, hold or play with baby†	132/135 (98.3%)	85/147 (58.9%)	97/102 (94.3%)	117/121 (96.5%)	68.8 (18.5, 256.1)	<0.0001
Hold or soothe baby at night†	131/135 (96.5%)	136/147 (92.9%)	98/103 (94.3%)	114/121 (94.0%)	1.9 (0.2, 14.5)	0.541
Take baby to health facility if sick†‡	65/133 (51.8%)	33/131 (25.4%)	45/84 (50.7%)	51/71 (73.1%)	9.1 (2.6, 31.4)	<0.0001

aOR – adjusted odds ratio, ANC – antenatal care

*Data are n (%) or n/N (%), with variance estimation accounting for health facility catchment clustering. Probability values from Wald χ^2^ tests based on cluster robust standard errors. aOR reflects a time by intervention group interaction, adjusted for individual men’s age, number of children, and educational attainment.

†Outcomes are sometimes/usually vs never.

‡Outcomes are yes vs no.

§Excludes men who reported their baby had never been sick.

No difference in effect on mean EPDS was observed between women who usually reside with their current male partner and those who do not (aRR = 1.13; 95% CI = 0.60, 2.12, *P* = 0.705), or between women who provided consent for their male coparent to complete the questionnaire and those who did not (aRR = 0.66; 95% CI = 0.40, 1.09, *P* = 0.102). Women whose male coparents completed the questionnaire had a greater reduction in mean EPDS compared with women whose male coparents did not complete the questionnaire (aRR = 0.40; 95% CI = 0.20, 0.81, *P* = 0.011).

No adverse events were detected. There was no effect detected on women subjected to physical intimate partner violence in the previous 12 months (aOR = 0.61; 95% CI = 0.36, 1.04, *P* = 0.070) or men experiencing controlling behaviours from their current female partner (*b* = 0.2; 95% CI = -3.4, 3.8, *P* = 0.898), with women experiencing reduced controlling behaviours from their current male partner as described in **Table 4**. No effect was detected on the proportion of women reporting they receive much too much (aOR = 0.93; 95% CI = 0.56, 1.52, *P* = 0.760) or much too little (aOR = 0.42; 95% CI = 0.09, 2.05, *P* = 0.283) support from their male coparent, or that it is always difficult to get support from their male coparent (aOR = 0.55; 95% CI = 0.15, 1.99, *P* = 0.361).

## DISCUSSION

To our knowledge, this is the first gender-synchronised, community-based intervention study of this kind. The intervention reduced symptoms of maternal depression and anxiety and increased care-seeking for MNCH services. These effects were detected together with increased women’s participation in household decision-making, decreased controlling behaviour and gender-inequitable attitudes among men, and increased practical support provided by men to their female coparents and babies during pregnancy, childbirth and up to six months after birth. While no data were captured on health outcomes beyond maternal mental health, observed improvements in uptake of couples’ HIV testing in pregnancy, timely postnatal care, and support for breastfeeding and maternal nutrition during and after pregnancy can be expected to improve MNCH outcomes [26], including by strengthening PMTCT in a context of high HIV prevalence [27]. Observed effects were achieved using a low-intensity, low-cost model, integrated with the health system and delivered through existing community-based health workers and monthly contacts by two project staff members.

Our findings support the study hypothesis (**Figure 1**) that a gender-synchronised intervention in this setting would influence men’s and women’s knowledge, attitudes, and behaviours in complementary ways to improve men’s support for MNCH, women’s participation in decision-making, and gender power dynamics in c-parent relationships, with positive impacts on MNCH care-seeking and maternal mental health.

The Mbereko+Men model brings together multiple interventions with demonstrated benefits for MNCH and mental health. Well-run women’s PLA groups facilitate mutual psychosocial support and health information sharing, and provide structured support for women’s participation in decision-making, to improve maternal mental health and MNCH care-seeking [3,4]. Problem-solving interventions using structured participatory activities to first identify barriers to participants’ desired outcomes, then plan to overcome these using existing resources, have improved women’s and men’s mental health in Zimbabwe [18]. Interventions increasing women’s control over cash and other resources have increased the uptake of MNCH services [28]. Male coparent support has previously been identified as an important and modifiable protective factor for maternal mental health [1], and interventions that influence men’s engagement in MNCH have improved maternal mental health [6], MNCH care-seeking [5], and PMTCT outcomes [27]. In the Mbereko+Men model, men’s discussion groups plausibly augmented the established benefits of PLA, problem-solving and microfinance interventions with women, by enabling men to participate in informed, equitable MNCH decision-making and provide improved support to women and babies. Indeed, thematic analysis of participatory charters for family health developed in men’s discussion groups, reported elsewhere [21], found these charters emphasised men’s commitments to sharing decision-making, supporting MNCH care-seeking, and contributing to unpaid domestic and care work. Furthermore, the rapid [29] community-level changes in men’s gender attitudes and supportive behaviours observed in this study suggest that gender-synchronised group discussions held separately with women and men, using family health and parenting roles during pregnancy and early childhood as an entry point, are a promising strategy to support gender norm change. Study findings also align with reviews highlighting the value of complementary activities with women and men to address gendered determinants of poor MNCH [9,30]. Our findings point to coparent relationships as a domain where the harmful impacts of entrenched gender inequality on MNCH outcomes [31] may be mitigated by targeting outcomes, such as decision-making, that are highly sensitive to gender power dynamics in the coparenting relationship [9,17,29].

The study implementation period was characterised by severe economic and political instability, as well as major political change shortly before the 2017 post-intervention survey. There was minimal disruption to intervention delivery or data collection since implementation was embedded within government community-based health systems with additional support from project staff as required. The rapidly changing context may explain the large secular decline in mean EPDS score and prevalence of EPDS≥12 between pre- and post-intervention.

The main limitation of the study is that the two-arm parallel design did not allow for the testing of the relative contribution of activities targeting women compared with those targeting men, or the additional impact of synchronising these components. However, literature identifying the value of gender-synchronised interventions [9,30], combined with the observed individual- and couple-level changes among men and women in this study, indicate that study findings document the impact of a gender-synchronised approach, which is plausibly greater than either the women-only or men-only components in isolation. The study was nested in a program with limited research-specific funding, and this influenced the study design; in particular, health facility catchment areas were selected as the clustering unit to align with the involvement of clinic staff in program implementation and limit travel costs. While this meant the intervention implementation was more similar to programs delivered at scale, a larger number of clusters would have increased statistical power. The study does not include data from eligible women who could not be reached during field research, or their male coparents. The study also does not include data from some male coparents of enrolled women: men whose enrolled female coparents did not consent to them completing the survey (n = 112), or men who were inaccessible during data collection (n = 209). It is not possible to assess how these women and men differ from those who completed the survey or determine any intervention effects on their behaviours and attitudes. Most outcomes in the hypothesised theory of change for the intervention (**Figure 1**) were measured, but some were not, and this limits our ability to infer how observed effects were achieved. The primary outcome of the study is measured through women’s self-report, and although multiple studies in Zimbabwe and elsewhere [1,10] confirm the validity of the EPDS measure, it is not possible to know whether participants misreported symptoms.

The non-normal distribution of EPDS scores, with a high proportion of 0 scores, was unexpected. Very low EPDS scores have been reported in contexts of chronic adversity, with a cut-off of 5/6 validated as indicating clinically significant symptoms of depression and anxiety in southwestern Nigeria [1]. The 11/12 cut-off previously validated in urban Zimbabwe [10] may, therefore, not be generalisable to the rural study population in Mutasa District. The difference in effect on mean EPDS between women whose male coparents participated in the survey compared with women who declined consent for their male coparent to participate, or whose male coparents were inaccessible, may indicate that the intervention is less effective for coparents experiencing relationship difficulties, despite the community-level effect on maternal mental health. This adds to the evidence that underlying relationship dynamics mediate the effect of coparent-focused interventions on maternal mental health [7].

The Mbereko+Men model is now recommended by the Government of Zimbabwe as a national community-based strategy for PMTCT [32]. Research is needed to assess the effectiveness and implementation fidelity of the intervention at scale, and particularly to explore the feasibility of synchronising the Mbereko and +Men components at scale. Future studies that engage fathers of young children are an important opportunity to assess paternal mental health using validated screening tools. Recruiting male survey participants through their female coparents provides an important safety mechanism for women. However, future research would capture community-level effects among men more accurately if men were recruited directly. This requires careful consideration of how to address sensitive topics such as intimate partner violence during data collection, as well as innovative strategies to directly reach fathers, including mobile men who engage in cross-border or seasonal work.

## CONCLUSIONS

Maternal mental morbidity and low uptake of MNCH services in resource-constrained settings are major contributors to the global burden of poor MNCH [2,26]. Maternal mental health and MNCH care-seeking are strongly influenced by gender-related social determinants of health, including gender differences in parenting roles and decision-making autonomy.[1,31] Study findings indicate that engaging women and men in separate, complementary reflections on gender-transformative health messages is an effective strategy to enhance men’s practical support for women and babies and enable coparents’ equitable, informed MNCH decision-making – and that these changes can improve maternal mental health and MNCH care-seeking in settings characterised by gender inequality and demand-side barriers to MNCH services. Our findings also demonstrate that low-intensity participatory group discussions with women and men in the same community, delivered using trained facilitators (including existing health workers), simple visual aids, and structured participatory activities, are a feasible, low-cost, acceptable approach for fostering rapid change in men’s and women’s attitudes, behaviours, and interactions within their coparent relationships. Many men and women in rural Zimbabwe and elsewhere value the opportunity for both parents to participate in MNCH [17,33]. If feasible and effective at scale, this gender-synchronised, low-intensity, community-based approach is a promising demand-side strategy to improve MNCH outcomes in resource-constrained settings.

## Figures and Tables

**Table:** EPDS – Edinburgh Postnatal Depression Scale, MNCH – maternal, newborn, and child health
